# Exploring nicotinamide cofactor promiscuity in NAD(P)H-dependent flavin containing monooxygenases (FMOs) using natural variation within the phosphate binding loop. Structure and activity of FMOs from *Cellvibrio* sp. BR and *Pseudomonas stutzeri* NF13

**DOI:** 10.1016/j.molcatb.2014.08.019

**Published:** 2014-11

**Authors:** Chantel N. Jensen, Sohail T. Ali, Michael J. Allen, Gideon Grogan

**Affiliations:** a York Structural Biology Laboratory, Department of Chemistry, University of York, Heslington, York YO10 5DD, UK; b Plymouth Marine Laboratory, Prospect Place, Plymouth PL1 3DH, UK

**Keywords:** Flavoprotein, Monooxygenase, FAD, Biotransformations, Sulfide

## Abstract

Flavin-containing monooxygenases (FMOs) catalyse asymmetric oxidation reactions that have potential for preparative organic synthesis, but most use the more expensive, phosphorylated nicotinamide cofactor NADPH to reduce FAD to FADH_2_ prior to formation of the (hydro)peroxy intermediate required for substrate oxygenation. A comparison of the structures of NADPH-dependent FMO from *Methylophaga aminisulfidivorans* (mFMO) and SMFMO from *Stenotrophomonas maltophilia*, which is able to use both NADPH and NADH, suggested that the promiscuity of the latter enzyme may be due in part to the substitution of an Arg–Thr couple in the NADPH phosphate recognition site in mFMO, for a Gln–His couple in SMFMO (Jensen et al., 2012, *Chembiochem*, 13, 872–878). Natural variation within the phosphate binding region, and its influence on nicotinamide cofactor promiscuity, was explored through the cloning, expression, characterisation and structural studies of FMOs from *Cellvibrio* sp. BR (CFMO) and *Pseudomonas stutzeri* NF13 (PSFMO), which possess Thr–Ser and Gln–Glu in the putative phosphate recognition positions, respectively. CFMO and PSFMO displayed 5- and 1.5-fold greater activity, respectively, than SMFMO for the reduction of FAD with NADH, and were also cofactor promiscuous, displaying a ratio of activity with NADH:NADPH of 1.7:1 and 1:1.3, respectively. The structures of CFMO and PSFMO revealed the context of the phosphate binding loop in each case, and also clarified the structure of the mobile helix–loop–helix motif that appears to shield the FAD-binding pocket from bulk solvent in this class of FMOs, a feature that was absent from the structure of SMFMO.

## Highlights

• Genes encoding ‘Type II FMOs’ CFMO and PSFMO were selected for diversity in the NADPH binding loop.
• CFMO and PSFMO were shown to both accept either NADH or NADPH as cofactor in the reduction of FAD.
• The activities with both NADPH and NADH have been evaluated in the oxidation of sulfide substrates.
• Structures of CFMO and PSFMO were solved, revealing the nature of the NADPH phosphate binding loop.

## Graphical abstract

**Figure:**
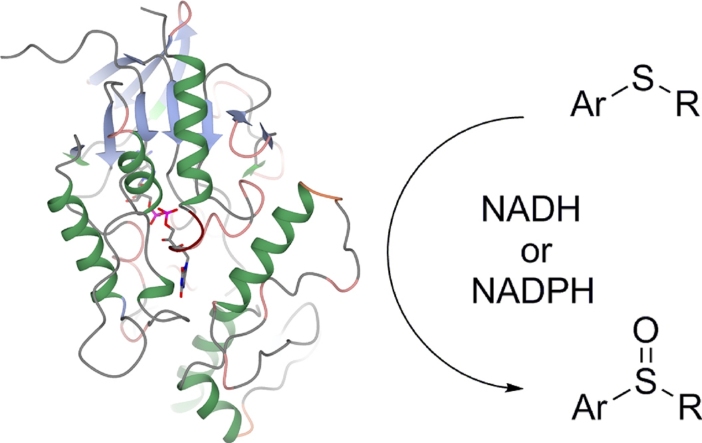

## 1 Introduction

Flavin-containing monooxygenases (FMOs) [1] catalyse the oxygenation of heteroatoms, such as nitrogen and sulfur, in various organic substrates, and have been studied both for their role in metabolism in higher eukaryotes, including humans [2], [3], and also for their contributions to microbial metabolism, in which they are able to catalyse the oxidation of amines [4] and amino acids such as ornithine [5]. In the case of microbial enzymes, the identification of interesting FMO activity has led to the exploitation of those enzymes for biotechnological applications [6], [7], in which the enzymatic characteristics of high turnover rates and chemo-, regio- and enantioselectivity, are very valuable. FMOs typically employ the phosphorylated nicotinamide cofactor NADPH to reduce a molecule of FAD within the enzyme, which then reacts with molecular oxygen to form a (hydro)peroxy flavin species that is the catalytic oxidant in reaction [1]. A well-studied example of a bacterial FMO is that from *Methylophaga aminisulfidivorans* (Uniprot Q83XK4, mFMO), an enzyme of monomer molecular weight 46 kDa that was identified on the basis of its ability to form the pigment indigo through oxidative transformation of indole [8]. mFMO has also been shown to catalyse the asymmetric sulfoxidation of a series of prochiral thioethers, when employed as part of a fusion enzyme with phosphite dehydrogenase for the recycling of the nicotinamide cofactor [9]. Studies of mFMO have shown a dependence for the phosphorylated cofactor NADPH, but the lower cost of the non-phosphorylated analogue, NADH, has meant that recent studies of FMOs have been directed towards enzymes that might employ that cofactor for flavin reduction.

We recently reported the cloning, expression and structural characterisation of another FMO, named SMFMO, from the marine bacterium *Stenotrophomonas maltophilia* [10]. This target was interesting as it displayed the ability to use either NADPH or NADH as the cofactor for reduction of the flavin. SMFMO was hence able to use NADH, along with a formate dehydrogenase/sodium formate based recycling system, to catalyse the asymmetric oxidation of thioethers, and also the Baeyer–Villiger oxidation of a strained, fused cyclobutanone substrate [10]. Related activities have recently been described by the group of Fraaije, who have characterised other FMOs, notably FMO-D, from *Rhodococcus jostii* RHA1, related to SMFMO, and which are similarly able to employ NADPH or NADH [11]. The structure of SMFMO was determined [10], and analysis of the nicotinamide cofactor binding loop revealed differences between NADPH-dependent mFMO [12], [13], [14] and SMFMO that might be significant in the recognition of the NADPH 2′ ribose phosphate that distinguishes NADPH and NADH [10]. In this region in mFMO, Arg234 and Thr235 project towards the phosphate and interact directly with the phosphate oxygen atoms, whereas in SMFMO Gln193 and His194 are found in equivalent positions. A double mutant of SMFMO that was designed to mimic the phosphate binding loop of mFMO, changed the preference of the enzyme for NADPH to NADH from a ratio of 1.5:1 to 1:3.5 [15]. These mutations were not successful in removing activity with NADH, however. Multiple studies on the wider group of NAD(P)H-dependent flavoprotein monooxygenases (FPMOs) have shown that, whilst positively charged basic residues are often involved in the specific recognition of negatively-charged phosphate [16], [17], [18], NADH-dependent activity can be engineered through the mutation of the cofactor binding loop to include a negatively charged carboxylate side chain enzymes that excludes phosphate, presumably through charge repulsion [19]. Engineering a glutamate residue into the cofactor-binding loop of SMFMO, in an attempt to generate a more NADH-specific variant, resulted in a mutant that was not produced in the soluble fraction of the *Escherichia coli* strain used for gene expression, however [15]. In this report, we describe the cloning, expression, and characterisation of two homologs of SMFMO, CFMO from *Cellvibrio* sp. BR (Uniprot code I3IEE4) and PSFMO from *Pseudomonas stutzeri* NF13 (M2V3J0). These homologs display natural variation in the cofactor-binding loop, Thr–Ser in CFMO and Gln–Glu in PSFMO, which suggested there may be altered cofactor preference compared to either mFMO or SMFMO. The enzyme activity with NADH and NADPH and a range of prochiral sulfides is assessed, and the structures of the enzymes, which reveal the context of the substituted amino acids within the putative cofactor binding loop, are presented.

## 2 Experimental

### 2.1 Chemicals

Chemicals, including media and buffer components, sulfide substrates and cofactors were purchased from Sigma-Aldrich (Poole U.K.).

### 2.2 Gene synthesis, cloning, expression and protein purification

The genes encoding CFMO and PSFMO were synthesised by GeneArt (Invitrogen), with sequences optimised for expression in *E. coli* using the GeneArt server program. Genes were then amplified by PCR from the commercial genes using the following primers: For CFMO: Forward: CCAGGGACCAGCAATGGATACACCGGTTATGG; Reverse: GAGGAGAAGGCGCGTTAGGCGCTATCCAGATACTG; For PSFMO: Forward: CCAGGGACCAGCAATGCCTCCGATTCTGG; Reverse: GAGGAGAAGGCGCGTTACGGACGACGGCTCGG. PCRs were analysed on agarose gels, and bands of the expected size were isolated using a PCR Cleanup kit^®^ (Qiagen). Target genes were then sub-cloned into the pET-YSBL-LIC-3C vector following a previously published procedure [20]. The recombinant plasmids were then used to transform cells of *E. coli* XL1-Blue (Novagen), which, after transformation and overnight growth on LB agar containing 30 μg mL^−1^ kanamycin as antibiotic marker, were subjected to miniprep procedures that resulted in plasmids suitable for DNA sequencing. Once the sequence of the genes had been confirmed, gene expression was conducted by transforming cells of *E. coli* BL21 (DE3) with the recombinant plasmids. 5 mL of LB medium containing 30 μg mL^−1^ kanamycin was inoculated with a single colony of the relevant strain. This starter culture was grown at 37 °C overnight with shaking at 180 r.p.m. Each 5 mL culture was then used to incolulate 500 mL LB broth containing 30 μg mL^−1^ kanamycin in a 2 L Erlenmeyer flask. These larger cultures were grown with shaking at 37 °C until the optical density, as determined by measurement at 600 nm, had reached 0.8. The cultures were then induced through the addition of 1 mM isopropyl β-D-1-thiogalactopyranoside (IPTG), and growth continued at 18 °C overnight. Cells were then harvested by centrifugation for 15 min at 4225 g using a Sorvall GS3 rotor in a Sorvall RC5B Plus centrifuge. Following centrifugation, the resultant cell pellets were resuspended in 25 mL 50 mM Tris/HCl buffer pH 7.5, containing 300 mM sodium chloride (‘buffer’) per L of cell growth. These suspensions were then subjected to cell disruption using an ultrasonicator for 3 × 30 s periods at 4 °C with intervals of 1 min. The soluble fraction after sonication was recovered by centrifuging the suspension for 30 min at 26,892 g in a Sorvall SS34 rotor. Supernatants were then filtered using a 2 μm Amicon filter, and then subjected t nickel affinity chromatography using a 5 mL His-Trap™ Chelating HP column. After loading the filtered protein solution, the column was washed with five column volumes of buffer containing imidazole (30 mM). The FMOs were then eluted from the column using a 30–500 mM imidazole gradient over twenty column volumes. Column fractions containing FMOs were identified using SDS-PAGE and combined. Pooled fractions were concentrated, typically, to a volume of 4 mL using a Centricon^®^ filter membrane (10 kDa cut-off) and 2 mL of this solution then loaded onto an S75 Superdex™ 16/60 size exclusion column that had been pre-equilibrated with buffer. FMOs were eluted with buffer at a flow rate of 1 mL min^−1^. Fractions were analysed by SDS-PAGE and those that contained pure FMOs pooled and stored at 4 °C for crystallisation, enzyme assays or biotransformations. For the purposes of crystallisation, the histidine tags of CFMO or PSFMO were cleaved using 3 C protease and using a procedure described previously [20]. Typical CFMO and PSFMO preparations yielded 20 mg and 7.5 mg pure protein per litre of cells, respectively.

### 2.3 Enzyme assays

Steady-state kinetic constants for the NADH and NADPH-dependent reduction of FAD by the FMOs were determined using the method employed previously [10], [21]. In a 1 mL quartz cuvette containing Tris–HCl buffer pH 7.5 (50 μmol) the decrease in absorbance at 340 nm was monitored for concentrations of NAD(P)H (10–100 μM) after the addition of enzyme (CFMO or PSFMO, 3.9 nmol). All data points represented the average of three separate runs. Kinetic constants (*K*_*M*_ and *k*_cat_) were calculated using a value for *ɛ* of 6220 mol dm^−3^ cm^−1^ using Grafit™ (Erithacus Sofware).

### 2.4 Biotransformations

Biotransformations using isolated enzymes with cofactor recycling were performed using the method previously described for SMFMO [10]. For NADH-dependent biotransformations: To a 10 mL round bottomed flask containing Tris–HCl buffer pH 7.5 (5 mL) were added substrate(s) (**1**–**6** in 100 μL ethanol to a final concentration of 5 mM), NADH (5 mg, a final concentration of 0.7 mM), formate dehydrogenase (5 mg), sodium formate (6.8 mg, 0.1 mmol) and CFMO or PSFMO (1 mL of a 5 mg mL^−1^ solution, 0.13 μmol). The reactions were then stirred for 24 h at room temperature. Aliquots (500 μL) were taken at intervals and extracted with ethyl acetate (500 μL). The organic layer was transferred to a GC vial and analysed by GC as described previously [10]. For NADPH-dependent biotransformations: To a 10 mL round bottomed flask containing Tris–HCl buffer pH 7.5 (5 mL) were added substrate(s) (**1**–**6** in 100 μL ethanol to a final concentration of 5 mM), NADPH (5.7 mg, a final concentration of 0.7 mM), glucose-6-phosphate-dehydrogenase (0.14 mg), glucose-6-phosphate (5.2 mg, 0.02 mmol) and CFMO or PSFMO (1 mL of a 5 mg mL^−1^ solution, 0.13 μmol). The reactions were then stirred for 24 h at room temperature and organic extracts of 500 μL aliquots analysed as previously [10]. Chiral analysis of sulfoxide products was carried out using BGB 173 and BGB-175 columns (30 m × 0.25 mm × 0.25 μm; each from BGB-Analytik) according to procedures described previously [10].

### 2.5 Protein crystallisation

Pure PSFMO and CFMO was subjected to crystallisation trials using a range of commercially available screens in 96-well plates employing 300 nL drops at a range of protein concentrations (3, 10 and 20 mg mL^−1^). The best crystals for PSFMO were obtained using the Clear Strategy Screen (CSS) [22] conditions containing 35% (w/v) tacsimate pH 7.0 and His_6_-tag cleaved protein at a concentration of 20 mg mL^−1^. The best crystals for CFMO were obtained using the CSS conditions containing 1.5 M ammonium sulfate and non-cleaved protein at 20 mg mL^−1^. Larger crystals for diffraction analysis using optimised conditions were prepared using the hanging-drop vapour diffusion method in 24-well plate Linbro dishes and using crystallisation drops of 2 μL, comprised of 1 μL of reservoir solution and 1 μL of protein solution at 20 mg mL^−1^. For PSFMO, the best crystals were again obtained in crystal drops containing 35% (w/v) tacsimate at pH 7.0 with no further additions. For CFMO, the best crystals were obtained in crystal drops containing 1.5 M ammonium sulfate and 1% propan-2-ol (v/v) at pH 7.0. Crystals were flash-cooled in a cryogenic solution containing the mother liquor with 10% (v/v) glycerol, and tested for diffraction using a Rigaku Micromax-007HF generator fitted with Osmic multilayer optics and a MARRESEARCH MAR345 imaging plate detector. Crystals that diffracted to a resolution of greater than 3 Å were retained for full dataset collection at the synchrotron.

### 2.6 Data collection, structure solution, model building and refinement of CFMO and PSFMO

Datasets described herein were collected at the Diamond Light Source, Didcot, Oxfordshire, U.K. Data for CFMO and PSFMO were each collected on beamline I24. Data were processed and integrated using XDS [23] and scaled using SCALA [24] included in the Xia2 processing system [25]. Data collection statistics are given in Table 1. The crystals of CFMO were in space group *C*2. The structure of CFMO was solved using MOLREP [26], using a monomer model of SMFMO (PDB code 4a9w) [10]. The solution contained two molecules in the asymmetric unit, representing one dimer, and the solvent content was 55%. The crystals of PSFMO were in space group *P*3_2_21. The structure of PSFMO was again solved using SMFMO as a model, but in this case, the solution contained only one molecule in the asymmetric unit. The solvent content in this case was 57%. The structures of CFMO and PSFMO were built and refined using iterative cycles using Coot [27] and REFMAC [28], employing local NCS restraints in refinement. The final structures exhibited *R*_cryst_ and *R*_free_ values of 24.6 and 28.7 (CFMO) and 16.2 and 20.4% (PSFMO), respectively. Each structure was validated prior to deposition using PROCHECK [29]. Refinement statistics for all structures are presented in Table 1. The Ramachandran plot for CFMO showed 93.4% of residues to be situated in the most favoured regions, 6.2% in additional allowed and 0.5% residues in outlier regions. For PSFMO, the corresponding values were 92.8%, 4.6% and 2.6%, respectively. The coordinates and structure factors for CFMO and PSFMO have been deposited in the Protein Data Bank with the accession codes 4usq and 4usr, respectively.

**Table 1:** Data collection and resolution statistics for CFMO and PSFMO. Values for the highest resolution shells are given in parentheses.

	CFMO	PSFMO
Beamline	Diamond i24	Diamond i24
Wavelength (Å)	0.96862	0.96862
Resolution (Å)	22.44–2.39 (2.45–2.39)	35.94–1.83 (1.88–1.83)
Space group	*C*2	*P*3_2_21
Unit cell	*a* = 115.41; *b* = 95.09; *c* = 92.37	*a* = *b* = 63.56; *c* = 189.82
	*α* *=* *β* = 90.0; *γ* *=* 126.3	*α* *=* *β* *=* 90.0; *γ* = 120.0
No. of molecules in the asymmetric unit	2	1
Unique reflections	31344 (2329)	41893 (3176)
Completeness (%)	98.1 (98.4)	100 (99.9)
*R*_merge_ (%)	0.09 (0.50)	0.112 (0.62)
*R*_p. i. m._	0.09 (0.50)	0.054 (0.30)
Multiplicity	3.2 (2.9)	9.8 (9.9)
〈*I/σ*(*I*)〉	9.0 (2.1)	14.9 (3.8)
CC_1/2_	0.99 (0.76)	1.00 (0.90)
Overall *B* factor from Wilson plot (Å^2^)	25	13
*R*_cryst_/*R*_free_ (%)	24.6/28.7	16.2/20.4
r.m.s.d. 1–2 bonds (Å)	0.014	0.02
r.m.s.d. 1–3 bonds (°)	1.66	2.26
Avge main chain B (Å^2^)	38	19
Avge side chain B (Å^2^)	40	22
Avge water B (Å^2^)	31	24

## 3 Results and discussion

### 3.1 Target selection

**Fig. 1:**
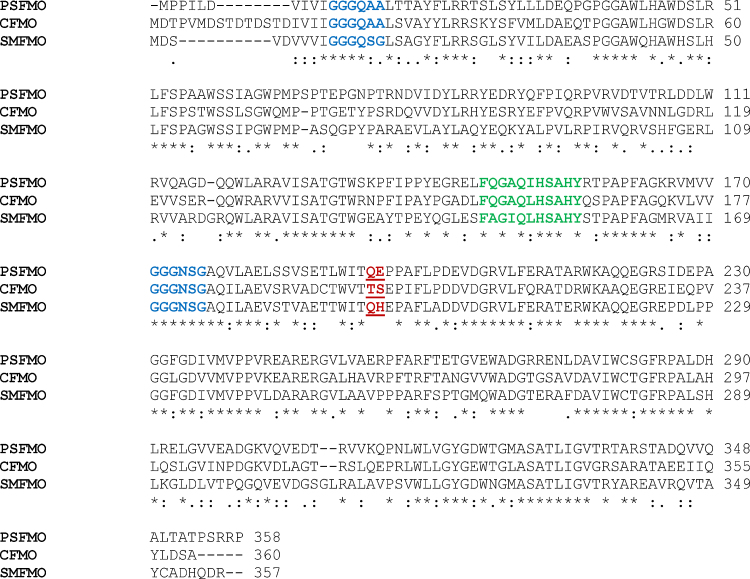
Sequence alignment of SMFMO, CFMO and PSFMO. The characteristic Rossman motifs are highlighted in blue; the FMO motif [30] in green, and the two residues proposed to be the closest to the 2′ ribose hydroxyl phosphate in red. (For interpretation of the references to color in this figure legend, the reader is referred to the web version of this article.)

Following the failure to incorporate a glutamate residue within the cofactor binding loop of SMFMO that might engender discriminatory binding of NADH over NADP in that enzyme, it was decided to select targets from the genomic databanks representative of natural variation within that loop, particularly corresponding to the Arg–Thr and Gln–His couples of mFMO of SMFMO, respectively. It was hoped that these variants might be sufficiently different within the loop to display a shift in preference for one nicotinamide cofactor, preferably NADH, over NADPH. CFMO (Uniprot code I3IEE4) and PSFMO (M2V3J0) were selected on this basis. Each is a putative FMO of a similar size to SMFMO (361 and 358 amino acids, respectively, *versus* 357 for SMFMO) and contains two Rossman domains and the FXGXXXHXXXY FMO motif [30]. A sequence alignment of these targets and SMFMO (Fig. 1) revealed 58% and 61% sequence identity between CFMO and SMFMO and PSFMO and SMFMO, respectively. In place of the Arg234Thr235 couple in mFMO or Gln193His194 couple in SMFMO, CFMO possessed Ser202 and Thr203, and PSFMO, Gln194 and Glu195, respectively. The latter was particularly interesting in being one of the only homologs identified from database searches as having either a glutamate or aspartate residue within the putative phosphate recognition region. No homolog was identified yet that possessed a Glu or Asp residue in place of Gln193 in SMFMO. Genes encoding CFMO and PSFMO were synthesised, subcloned and expressed in the soluble fractions of transformed strains of *E. coli* BL21 (DE3). The proteins were readily purified using nickel affinity and size exclusion chromatography as described in Section 2, each yielding protein solutions of a bright yellow colour, indicative of the presence of bound FAD.

### 3.2 Cofactor promiscuity in the reduction of FAD

The ability of CFMO and PSFMO to use either NADH or NADPH to reduce FAD was assessed using standard UV spectrophotometry assays [10], [21], in which the oxidation of NAD(P)H was monitored at 340 nm. Kinetic parameters (*K*_m_ and *k*_cat_) were determined for each enzyme and each cofactor and the results are shown in Table 2, with data for SMFMO [10] reproduced for comparison.

**Table 2:** Kinetic constants for CFMO and PSFMO in the reduction of flavin by either NADH or NADPH. Values for SMFMO, reproduced from Ref. [10] are also given for comparison.

Enzyme/cofactor	*K*_M_ (μM)	*k*_cat_ (s^−1^)	*k*_cat_/*K*_M_ (M^−1^ s^−1^)
SMFMO*	24 ± 9	0.03	1250
NADH			
SMFMO	27 ± 5	0.02	740
NADPH			
CFMO	3 ± 1	0.02	6670
NADH			
CFMO	5 ± 1	0.02	4000
NADPH			
PSFMO	16 ± 2	0.03	1880
NADH			
PSFMO	17 ± 2.0	0.04	2350
NADPH			

* Data taken from Ref. [10].

The first observation was that both CFMO and PSFMO were both able to accept NADH or NADPH for the reduction of FAD. CFMO was observed to bind both NADH and NADPH with increased affinity compared to SMFMO, as illustrated by *K*_m_ values of 3 and 5 μM compared to values in the range of 24–27 μM for NADH and NADPH, respectively. Although *k*_cat_ values were lower for CFMO than the other two enzymes, the low *K*_m_ contributed to CFMO having a catalytic activity, as determined by values of *k*_cat_/*K*_M_, of 5.3 and 5.4 fold greater than SMFMO for NADH and NADPH, respectively. These values also reveal a slight preference for NADH by a factor of 1.7, compared with SMFMO, for which the value was 1.5. For PSFMO, the *K*_M_ was similar with either NADH (16 μM) or NADPH (17 μM) which were each lower than for SMFMO. *k*_cat_/*K*_M_ was slightly lower with NADH (1880 M^−1^ s^−1^) than NADPH (2350 M^−1^ s^−1^), indicating a preference for NADPH in this case of 1.3, but each value was again higher than that for SMFMO with either cofactor.

### 3.3 Biotransformations of prochiral sulfides using CFMO and PSFMO using either NADH or NADPH as cofactor

**Fig. 2:**
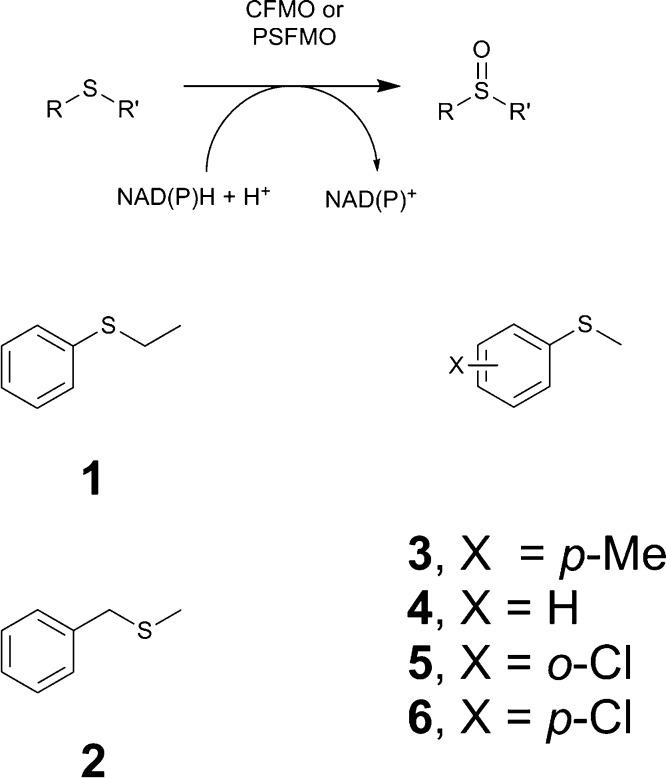
Sulfide substrates screened in this study against CFMO and PSFMO.

Both CFMO and PSFMO were then challenged with a range of prochiral sulfide substrates **1**–**6** (Fig. 2), with the addition of either catalytic NADH or NADPH as cofactor, and in the presence of a suitable cofactor recycling system, as had been used for SMFMO [10]. The results of all biotransformations by CFMO and PSFMO, compared to those obtained with SMFMO are shown in Table 3. CFMO oxidised sulfides **1** and **3–6** to, for the most part, their corresponding (*R*)-sulfoxides, with either cofactor. However, substrate **1** was transformed to the (*S*)-sulfoxide in the presence of NADPH. The conversions were overall higher with NADH, however, the enantiomeric excess of sulfoxide products was slightly higher with NADPH in general. Substrate **2** was not converted by CFMO in the presence of either cofactor. The highest conversion was seen for substrate **3**, methyl tolyl sulfide, with 65% for NADH (22% e.e. (*R*)-sulfoxide product) and 32% for NADPH (32% e.e.). The most enantioselective transformation was that of substrate **6** with 66% -(*R*) and 77% -(*R*) for achieved with NADH or NADPH, respectively. CFMO was overall less active than SMFMO with sulfide substrates and NADH, although both the conversion and product enantiomeric excess in the oxidation of phenyl methyl sulfide **4** were superior. Interestingly, when NADPH was employed as nicotinamide cofactor CFMO gave higher conversions compared to SMFMO, except for substrate **3,** possibly indicative of its greater activity as shown by the kinetic constants. For PSFMO, the highest conversions were observed for substrate **3** and **4**, but enantioselectivity was poor in each case. PSFMO again gave (*R*)-sulfoxide products for the most part. The most enantioselective reaction was observed for substrate **6**, yielding (*R*)-sulfoxide product of 85% and 57% with NADH and NADPH, respectively. Overall, PSFMO catalysed sulfoxidation reactions gave higher conversions than either SMFMO or CFMO, but with poorer e.e.s overall, save for substrate **6**.

**Table 3:** Results of biotransformations of prochiral sulfide substrates **1** and **3–6** by CFMO or PSFMO using either NADH or NADPH as nicotinamide cofactor. Values for SMFMO, reproduced from Ref. [10] are also given for comparison.

Sulfide	NADH			NADPH		
	Conversion (%); absolute configuration; e.e.			Conversion (%); absolute configuration; e.e. (%)		
	SMFMO*	CFMO	PSFMO	SMFMO*	CFMO	PSFMO
1	27, (*R*)-, 71% e.e.	17, (*R*)-, 54% e.e.	61, (*R*)-, 30% e.e.	2, (*R*)-, 57% e.e.	6, (*S*)-, 43% e.e.	28, (*R*)-, 24% e.e.
3	90, (*R*)-, 25% e.e.	65, (*R*)-, 22% e.e.	97, (*R*)-, 47% e.e.	33, (*R*)-, 44% e.e.	32, (*R*)-, 32% e.e.	78, (*R*)-, 32% e.e.
4	8, (*R*)-, 21% e.e.	64, (*R*)-, 58% e.e.	99, (*R*)-, 14% e.e.	1, n.d.	38, (*R*)-, 64% e.e.	73, (*S*)-, 4% e.e.
5	6, (*S*)-, 15% e.e.	No conversion	13, (*S*)-, 10% e.e.	<1, n.d.	11, (*R*)-, 3% e.e.	No conversion
6	40, (*R*)-, 80% e.e.	14, (*R*)-, 66% e.e.	50, (*R*)-, 85% e.e.	9, (*R*)-, 82% e.e.	47, (*R*)-, 77% e.e.	22, (*R*)-, 57% e.e.

* Data taken from Ref. [10]. n.d. = not determined.

### 3.4 Structures of CFMO and PSFMO

**Fig. 3:**
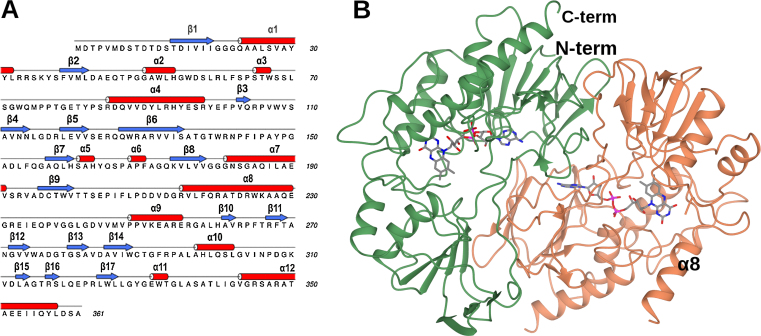
(a) Primary structure of CFMO, with secondary structure assignment, created using DSSP [32], [33] and represented using ALINE [34]. (b) Structure of dimer of CFMO, in ribbon format, with subunit **A** in green and **B** in coral. One FAD molecule per monomer is shown in cylinder format with carbon atoms in grey. Helix α8, which forms part of the flexible helix–loop–helix structure that may shield the active site from bulk solvent, is also highlighted. (For interpretation of the references to color in this figure legend, the reader is referred to the web version of this article.)

In order to shed light on the nature of the cofactor binding loops in CFMO and PSFMO, the structures of each in complex with the flavin FAD were determined to resolutions of 2.39 Å and 1.83 Å, respectively. Crystals of CFMO grew in the *C*2 space group, with two molecules ‘A’ and ‘B’, representing one dimer in the asymmetric unit. The CFMO dimer was made up of two monomers (Fig. 3b), sharing an interfacial area of approximately 1207 Å^2^. Analysis of the CFMO structure using PISA [31] found that the interactions that stabilise the dimer included six hydrogen bonds, including those between the backbone nitrogen of Trp108(A) and the side chain oxygen of Glu124(B) and the side chain N—H of Gln320(A) with the backbone carbonyl of Ile145(B). The calculated dissocation energy of the dimer interface (Δ^i^*G*) was −4.4 kcal mol^−1^. The monomer of CFMO superposed with the structure of the SMFMO monomer with an r.m.s.d. of 0.77 Å over 317 Cα atoms. The secondary elements of the structure are summarised in Fig. 3a. Each CFMO monomer consists of two domains, an FAD binding domain and the putative substrate binding domain. Electron density was visible for the majority of the backbone in each monomer, from residue Ser13 to Ala361, with a stretch of missing density corresponding to six amino acids between positions Gln236 and Asp243 (PVGGLG) in subunit A and fifteen between Ala227 and Asp243 (AQEGREIEQPVGGLG) in subunit B that could not be modelled. Side chain density in the region of residues 224–236 in subunit A was also missing. It appears that the residues for which there is poor electron density are part of a flexible helix–loop–helix structure, incorporating helix α8, which is found over the FAD binding pocket, perhaps shielding the active site from bulk solvent. An equivalent region of density was missing in structures of both wild-type SMFMO (4A9W) [10] and the Gln193Arg/His194Thr mutant (4C5O) [15]. There was substantial residual density in the omit map in the putative active site following building and refinement of the protein atoms of CFMO. This was successfully modelled and refined as FAD in the flat, oxidised form.

**Fig. 4:**
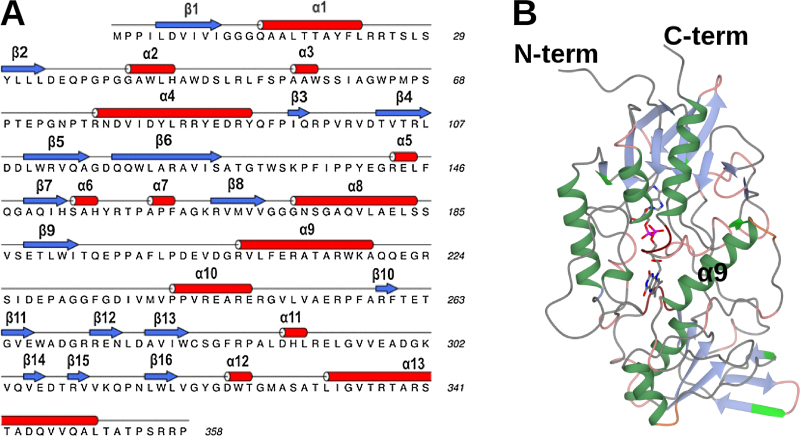
(a) Primary structure of PSFMO, with secondary structure assignment, created using DSSP [32], [33] and represented using ALINE [34]. (b) Structure of PSFMO monomer, in ribbon format. One FAD molecule per monomer is shown in cylinder format with carbon atoms in grey. Helix α9, which with helix α10 forms part of the flexible helix–loop–helix structure over the active site, and for which electron density is continuous in the PSFMO structure, is also highlighted.

Crystals of PSFMO grew in the *P*3_2_21 space group, with one molecule in the asymmetric unit, although both size-exclusion studies (not shown), and the relationship of the monomer to its closest crystallographic symmetry partner, are strongly suggestive of an active dimer for PSFMO as with SMFMO and CFMO. Analysis of a dimer pair by PISA [31] revealed an interfacial area of 955 Å^2^, with six H-bonds and 8 salt bridges formed between the monomers. The calculated dissociation energy of the dimer interface (Δ^i^*G*) was −7.1 kcal mol^−1^. The secondary structural elements are summarised in Fig. 4a and the monomer structure is shown in Fig. 4b. In the case of PSFMO, there was electron density for each amino acid in the monomer from residue Met1 to Pro354, which allowed for the first time the observation of the entire backbone of the flexible helix–loop–helix structure, represented by residues 207–234 in PSFMO, and incorporating helices α9 and α10, over FAD that had been missing from SMFMO structures and only partially present in CFMO, although side-chain density for residues 222–227 was poor. Electron density was also present for residues Gly-2, Pro-1 and Ala 0 at the N-terminus, which represent the first part of the linker to the His-tag in the protein produced using the YSBLIC-3C construct. The monomer of PSFMO superposed with the structure of the SMFMO with an r.m.s.d. of 0.90 Å over 327 Cα atoms. FAD was again clearly visible in the omit map after building and refinement of the protein atoms, and was also modelled and refined successfully in the oxidised form, the density revealing no puckering of the tricyclic ring system that might be indicative of the reduced flavin.

### 3.5 The FAD binding region in CFMO and PSFMO

**Fig. 5:**
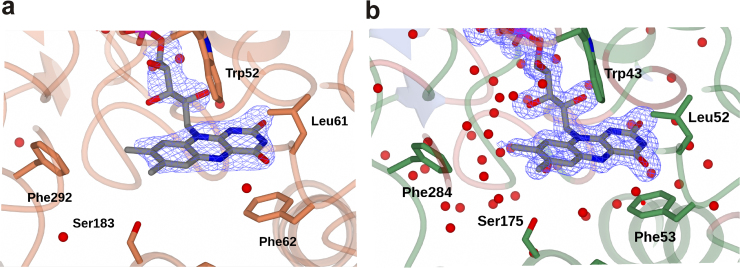
FAD environment within the active sites of (a) CFMO and (b) PSFMO. The peptide backbones are shown in with transparency levels of 30%. Amino acid side chains within the active site are shown in cylinder format in coral for CFMO and green for PSFMO. The FAD molecules are shown in cylinder format with carbon atoms in grey, surrounded by the *F_o_* − *F_c_* omit map in blue displayed at a level of 3*σ* in each case, which was obtained through refinement in the absence of FAD. The maps illustrate the superior quality of the resolution (1.83 Å vs. 2.39 Å) in the PSFMO structure.

In both CFMO and PSFMO, the tricyclic isoalloxazine ring of FAD was bound at the domain interface in a cavity beneath the flexible helix–loop–helix region of each enzyme (Fig. 5a and b). The oxygen atoms of the pyrimidinedione ring form H-bonds with the backbone N—Hs of CFMO Phe62 (PSFMO Phe53) and Leu340 (Leu322). In common with SMFMO, the aromatic side-chain of a phenylalanine residue CFMO Phe 62 (PSFMO Phe53) is found beneath the pyrimidinedione ring in the place where, in mFMO, an asparagine residue is found, which is thought to stabilise the oxygenating species in catalysis. Mutation of this Asn to a serine residue in mFMO led to removal of oxygenase activity [13], but in SMFMO, mutation of the Phe residue to a valine resulted in a mutant of inverted enantioselectivity for the transformation of *para*-tolyl methyl sulfide [15]. The similarity in the FAD environment in all three homologs is reflected in the broadly similar substrate ranges and enantioselectivities of the cofactor-promiscuous enzymes with (*R*)-selectivity conserved for the most part towards substrates **1**, **3**, **4** and **6** with NADH. The poorer performance of NADPH-dependent biotransformations by SMFMO, CFMO and PSFMO appears at variance with their comparable abilities to reduce FAD, but may be due to more effective participation of the NADH cofactor in stabilisation of the active site during oxygenation, as has been observed for mFMO [12].

### 3.6 Cofactor binding loops in CFMO and PSFMO

**Fig. 6:**
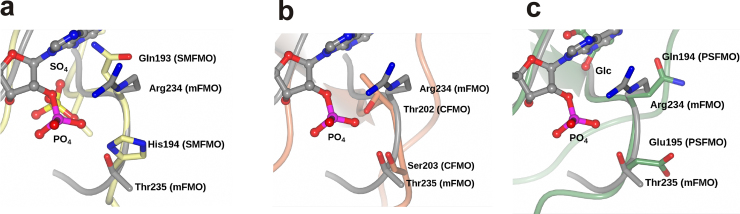
The putative NADP^+^ ribose 2′ phosphate recognition site in (a) SMFMO, (b) CFMO and (c) PSFMO, each superimposed with the corresponding site in mFMO, derived from PDB accession code 2XLT. The backbone and side chain and NADP^+^ carbon atoms for mFMO are shown in grey in each case. Glc = glycerol in (c). Equivalent features for SMFMO, CFMO and PSFMO are shown in yellow, coral and green in (a), (b) and (c) respectively. (For interpretation of the references to color in this figure legend, the reader is referred to the web version of this article.)

While the structures of SMFMO [10], [15] have not yet been determined in complex with a nicotinamide cofactor, superimposition with the structure(s) of NADPH-complexes of mFMO (*e.g.* 2XLT) revealed that the phosphate binding sites for NADPH, including the 2′ hydroxyl ribose phosphate that distinguishes NADPH from NADH, were occupied by sulfate ions that had resulted from the crystallisation conditions in lithium sulfate (Fig. 6a). This observation led to the hypothesis that substitution of arginine and threonine residues in mFMO for glutamine and histidine in SMFMO were one of the factors that determined cofactor promiscuity in the latter enzyme. Having acquired the structure of CFMO and PSFMO, these were now superimposed with the structure of mFMO in order to gain insight into the changes in the cofactor binding loops that may occur as a result of having threonine/serine and glutamine/glutamate in these positions. In the case of CFMO the side-chain oxygen atoms of Thr202 and Ser203 are both orientated towards the putative phosphate binding site (Fig. 6b). The structure(s) of mFMO had previously demonstrated that threonine is able to make an effective contact with one of the phosphate oxygen atoms of NADPH [12], [13], [14].

The nucleotide binding site of PSFMO featured a glycerol molecule, from the cryoprotectant, occupying approximately the same position as the adenine ring of NADPH in mFMO, and with the hydroxyl groups forming hydrogen bonds with the side chain of Tyr140, the backbone NH of Gln194 and a water molecule. Superimposition of the putative phosphate binding sites of SMFMO and PSFMO revealed that the backbone Cα atoms of residues thought to be responsible for cofactor promiscuity in SMFMO, Gln193 and His194, are in the same position as those in PSFMO, Gln194 and Glu195 (Fig. 6c). However, the side chains of both Gln194 and Glu195 are observed to point away from the putative phosphate binding site into the bulk solvent.

No data on the activity with mFMO and NADH have been reported, but other NADPH-specific FPMOs, such as NADPH-dependent Baeyer–Villiger monooxygenases possess the Arg–Thr couple in common with mFMO [16], [17]. The studies with SMFMO and with CFMO and PSFMO reported herein suggest first that a Gln–His couple favours NADH binding slightly, but that a Thr–Ser couple, as found in CFMO is superior for activity with both cofactors overall. Counter-intuitively, the presence of a glutamate in the second position of the couple, as found in PSFMO, does not prohibit NADPH binding, but rather the Glu side chain is able to project away from the putative phosphate binding site. It is possible that the presence of the glutamate side-chain allows selective recognition of the NADH ribose oxygen atoms in the presence of that cofactor, by rotating into the binding pocket, but is able to rotate away in the presence of NADPH, thus allowing cofactor promiscuity in PSFMO. More detailed analysis awaits the acquisition of structures of these enzymes in the presence of both NADPH and NADH however.

## 4 Conclusion

CFMO and PSFMO provide new examples of enzymes within the emerging sub-family of cofactor-promiscuous flavin-containing monooxygenases, named ‘Type II FMOs’ by Fraaije and co-workers [7], [11]. The study of this sub-class of FMOs is providing new information on structure and evolutionary diversity within this family of enzymes, and also suggesting new avenues for the engineering of related enzymes for cofactor promiscuity with a view to greater suitability for application in biocatalytic processes.
